# The Enhanced *In Vivo* Activity of the Combination of a MEK and a PI3K Inhibitor Correlates with [^18^F]-FLT PET in Human Colorectal Cancer Xenograft Tumour-Bearing Mice

**DOI:** 10.1371/journal.pone.0081763

**Published:** 2013-12-10

**Authors:** Emma J. Haagensen, Huw D. Thomas, Ian Wilson, Suzannah J. Harnor, Sara L. Payne, Tommy Rennison, Kate M. Smith, Ross J. Maxwell, David R. Newell

**Affiliations:** 1 Newcastle Cancer Centre, Northern Institute for Cancer Research, Paul O'Gorman Building, Medical School, Newcastle University, Framlington Place, Newcastle-upon-Tyne, United Kingdom; 2 Newcastle Cancer Centre, Northern Institute for Cancer Research, School of Chemistry, Bedson Building, Newcastle University, Newcastle, United Kingdom; University of South Alabama Mitchell Cancer Institute, United States of America

## Abstract

Combined targeting of the MAPK and PI3K signalling pathways in cancer may be necessary for optimal therapeutic activity. To support clinical studies of combination therapy, 3′-deoxy-3′-[^18^F]-fluorothymidine ([^18^F]-FLT) uptake measured by Positron Emission Tomography (PET) was evaluated as a non-invasive surrogate response biomarker in pre-clinical models. The *in vivo* anti-tumour efficacy and PK-PD properties of the MEK inhibitor PD 0325901 and the PI3K inhibitor GDC-0941, alone and in combination, were evaluated in HCT116 and HT29 human colorectal cancer xenograft tumour-bearing mice, and [^18^F]-FLT PET investigated in mice bearing HCT116 xenografts. Dual targeting of PI3K and MEK induced marked tumour growth inhibition *in vivo*, and enhanced anti-tumour activity was predicted by [^18^F]-FLT PET scanning after 2 days of treatment. Pharmacodynamic analyses using the combination of the PI3K inhibitor GDC-0941 and the MEK inhibitor PD 0325901 revealed that increased efficacy is associated with an enhanced inhibition of the phosphorylation of ERK1/2, S6 and 4EBP1, compared to that observed with either single agent, and maintained inhibition of AKT phosphorylation. Pharmacokinetic studies indicated that there was no marked PK interaction between the two drugs. Together these results indicate that the combination of PI3K and MEK inhibitors can result in significant efficacy, and demonstrate for the first time that [^18^F]-FLT PET can be correlated to the improved efficacy of combined PI3K and MEK inhibitor treatment.

## Introduction

Numerous small molecule inhibitors of specific signal transduction pathways have been developed; in particular, the PI3K pathway, a major survival pathway, and the MAPK pathway, a major mitogenic pathway, have been targeted in cancer. However, single agent clinical activity with these inhibitors has in general been modest, and hence combinations are being evaluated [1]. Many combinations of PI3K and MAPK inhibitors have exhibited promising activity *in vitro* but some of the most impressive results have been seen *in vivo*.

As a single agent, the pan PI3K inhibitor GDC-0941 has modest preclinical *in vivo* efficacy, with dose-dependent activity over the range 25-150 mg/kg/day in the U87MG glioblastoma xenograft model [2]. Subsequently, doses of 75-150 mg/kg have been shown to result in tumour growth inhibition in a range of human tumour xenograft models including tumours that are *PIK3CA* mutant, *PTEN* null, *EGFR* mutant or wild type, with an associated decrease in AKT and S6 phosphorylation [2], [3], [4], [5], [6]. GDC-0941 displayed promising preclinical pharmacokinetics with good oral bioavailability (78% in mice), and on the basis of these data and the predicted pharmacokinetics in humans [2], [7], is now undergoing Phase I and II clinical trials as a single agent or in combination with chemotherapeutic agents [8], [9].

The allosteric MEK inhibitor PD 0325901 also exhibited promising selective pre-clinical anti-cancer efficacy *in vivo* as a single agent, doses of 10–25 mg/kg causing significant tumour growth inhibition and in many cases regression, in a range of murine and human tumour xenograft models, including those which were *BRAF* or *KRAS* wild type or mutant [6], [10], [11], [12], [13], [14]. Growth inhibition achieved with high doses of PD 0325901 was accompanied by a decrease in ERK1/2 phosphorylation, which was maintained even when lower doses of 1.5–3 mg/kg PD 0325901 were used; however, these lower doses were only able to cause a modest tumour growth delay [6], [10], [11], [12]. Oral and i.v. doses of PD 0325901 were shown to have comparable bioavailability, were non-toxic at <100 mg/kg, and resulted in a dose-dependent inhibition of ERK1/2 phosphorylation in rat liver and lungs due to inhibition of MEK [15]. However, clinical trials revealed that single agent PD 0325901 was associated with ocular and neurological toxicity, such as retinal vein occlusion [16], and thus clinical trials using single agent PD 0325901 have been terminated [8].

As the MEK inhibitor PD 0325901 appeared promising as a single agent but showed toxicity in clinical trials, and tumour growth inhibition was modest with the PI3K inhibitor GDC-0941 even at high doses, these and other PI3K and MEK inhibitors are now being investigated clinically in combination studies [8]. To this end, PD 0325901 is being studied in combination with the PI3K/mTOR inhibitor PF-04691502, and GDC-0941 is in a clinical trial in combination with the MEK inhibitor GDC-0973 [8].

*In vivo* pre-clinical studies have shown that combinations of PI3K and MEK inhibitors consistently result in improved tumour growth inhibition compared to either single agent, and in many cases cause regression in a variety of human tumour xenograft and mouse tumour models with a range of genetic backgrounds, including those with *KRAS*, *BRAF* and/or *PIK3CA* mutations, and/or *PTEN* deletions [6], [12], [17], [18], [19]. Furthermore, the responses observed with combination treatment were often durable, despite relatively low doses of both inhibitors being used in many studies. Combination of PI3K and MEK inhibitors have been shown to decrease the phosphorylation of S6, AKT and ERK1/2 [12], [19], and intermittent dosing studies have revealed prolonged effects on downstream markers of proliferation and apoptosis, such as a sustained decrease in cyclin D1 and an increase in Bim levels, which may be responsible in part for the improved response seen with the combination therapy [6], [19].

Pharmacodynamic biomarkers of MAPK and PI3K pathway modulation, such as those mentioned above, require repeated invasive biopsies and hence may not be clinically feasible. Furthermore, changes in tumour size or disease stabilisation, as measured by volumetric imaging methods such as CT and MRI, may not become apparent until after many weeks of therapy, which can delay clinical decision making and potentially result in patients inappropriately remaining on ineffective and toxic treatments for prolonged periods of time. To address the limitations of conventional volumetric imaging, positron emission tomography (PET) is being used in pre-clinical studies and clinical trials as a functional surrogate response imaging biomarker [13], [14].

The fluorine-modified thymidine analogue, 3′-deoxy-3′-[^18^F]-fluorothymidine ([^18^F]-FLT) is a PET radiotracer that is used for detecting anti-proliferative effects, as accumulation in cells is determined by the expression and activity of the enzyme thymidine kinase 1 and specific nucleoside transporters, both of which are under the control of S phase cell cycle regulators [13], [14], [20], [21], [22], [23]. Furthermore, the uptake of [^18^F]-FLT has been shown to correlate with standard proliferation markers, such as Ki67, TK1 and BrdU uptake [24], [25], [26], [27], [28], [29]. Using [^18^F]-FLT PET, changes in proliferation compared to baseline have been demonstrated in a variety of human tumour xenografts as early as 18, 24 and 120 hours using either single agent GDC-0941 or PD 0325901 [13], [14], [30], [31]. Additionally, [^18^F]-FLT PET has already been used to predict the efficacy of chemotherapy and radiotherapy [32], [33], [34] and recently 2 clinical trials have begun with the MEK inhibitor AZD6244 as a single agent incorporating [^18^F]-FLT PET [8], [35].

The aim of the studies described in this report was to determine whether [^18^F]-FLT PET can be used as a surrogate response biomarker for combined MEK and PI3K inhibitor therapy, as a prelude to clinical trials.

## Methods

### Ethics Statement

All experiments were reviewed and approved by the Newcastle University (UK) animal welfare committee, and were performed according to the guidelines for the welfare and use of animals in cancer research [36] and national law, under project license (PPL60/4442) issued by the UK Government Home Office under the animals (scientific procedure) act 1986.

### Cell Lines & Reagents

HCT116 and HT29 human colorectal cancer cells were obtained from the ATCC (American Type Culture Collection). All cell lines were grown in RPMI-1640 medium (supplemented with 10% (v/v) foetal bovine serum, 1% (v/v) penicillin (50 U/ml) – streptomycin (50 mg/ml) and 2 mM L-glutamine) and were confirmed free of mycoplasma contamination by regular testing with Mycoalert (Cambrex, Iowa, USA).

### Inhibitors

The MEK inhibitor PD 0325901 was kindly supplied by UCB Celltech, Slough, Berkshire, UK. The PI3K inhibitor GDC-0941 was either synthesised in house [37] or purchased from Stratech Scientific Ltd, Newmarket, Suffolk, UK. All batches of GDC-0941 were fully characterised using conventional chemical analyses, shown to be >99% pure, and generated biological results consistent with the authenticity of the compound. Both drugs were suspended in 0.5% hydroxypropyl-methylcellulose (w/v) and 0.2% Tween 80 (v/v) in sterile distilled water (MCT).

### Animals

Animal studies were all carried out using female athymic CD1 nude mice (Charles River, Kent, UK), implanted with HCT116 or HT29 xenografts (1×10^7^ cells in 50 µl media injected subcutaneously into the right flank), maintained and handled in isolators under specific pathogen-free conditions.

### Pharmacokinetic (PK) and Pharmacodynamic (PD) Studies

Mice bearing HCT116 human tumour xenografts were treated with either 1 mg/kg PD 0325901, 100 mg/kg GDC-0941 or the combination of 1 mg/kg PD 0325901 and 100 mg/kg GDC-0941, and were bled by cardiac puncture under terminal anaesthesia at selected time points post-treatment (0.25–24 hours, 3 mice/time point). Blood was collected into heparinised tubes, and plasma was separated and stored at −20°C until analysed. Tumours were removed, snap frozen in liquid nitrogen and stored at −80°C prior to PK and PD analyses, as described below.

For PK analyses, drug was extracted from 60 µl aliquots of samples by protein precipitation with 9 volumes of acetonitrile (MeCN). Samples were centrifuged at 3000g for 5 minutes at 4°C, and 500 µl of the supernatant evaporated to dryness under nitrogen gas at 30°C using a Zymark Evaporator (Caliper Life Sciences Limited, Cheshire, UK). Samples were reconstituted in 100 µl HPLC mobile phase consisting of 40% acetonitrile and 60% (v/v) 0.1% formic acid pH 4.0 (v/v), and 50 µl of the supernatant applied to a 10 cm Xterra Waters 186000436 C18 3.5 µm column (Waters, Hertfordshire, UK) fitted with an in line filter. Compounds were eluted with the above mobile phase at 1 ml/min using a Waters Millennium Chromatography system (Waters, Hertfordshire, UK). Analytes were detected by UV absorbance at 275 and 315 nm, at retention times of 6.8–7.3 and 3.9–4.3 minutes for PD 0325901 and GDC-0941, respectively. For analysis of drug in tumour tissue, tumours were homogenised in 3 volumes of PBS (w/v) and 50 µl aliquots extracted with 9 volumes of MeCN, and centrifuged, evaporated and analysed as described above. Total (free and protein bound) drug concentrations were determined using standard curves (0.1–10 µg/ml, r^2^>0.98 in all cases) generated by extracting compounds from the appropriate matrix, the extraction efficiency being >97% for all matrices. Paired t tests were used to compare the different treatment groups and differences with a p value <0.05 were considered statistically significant.

For PD analyses, tumours (2 to 4∼2.5 mm^3^ pieces) were disaggregated in 1 ml PhosphoSafe™ extraction reagent (Merck Chemicals Ltd, Nottingham, Nottinghamshire, UK) containing a protease inhibitor cocktail (Pierce, Thermo Scientific, Rockford, Illinois, USA) at the manufacturer's recommended dilution using a Medimachine™ (BD Biosciences, Oxford, UK), centrifuged, and the supernatant removed and analysed by Western blotting. Proteins were resolved on Novex® 4–12% (w/w) Tris-glycine gels (Invitrogen Ltd, Renfrew, Paisley, UK) and electrotransferred onto Hybond C nitrocellulose membrane (GE Healthcare Life Sciences, Hatfield, Hertfordshire, UK). Membranes were incubated with phospho-4EBP1 (Thr37/46) (#2855), phospho-p44/42 MAPK (Thr202/Tyr204) (#4370), phospho-Akt (Ser473) (#4060) or phospho-S6 ribosomal protein (Ser235/236) (#4858) monoclonal antibodies obtained from Cell Signalling Technology (New England BioLabs (UK) Ltd, Hitchin, Hertfordshire, UK). Antibody binding was detected by incubation with a HRP-conjugated goat anti-rabbit polyclonal antibody (Dako, Glastrop, Denmark). Blots were developed using Pierce ECL (enhanced chemiluminescence) western blotting substrate (Thermo Scientific, Rockford, Illinois, USA), or SuperSignal® West Dura extended duration substrate (Thermo Scientific, Rockford, Illinois, USA), and Kodak X-ray film (Genetic Research Instrumentation Ltd, Braintree, Essex, UK) on a MediPhot 937 film developer (Colenta, Weiner Neustadt, Austria), then digitally scanned.

### Determination of Anti-Tumour Activity

Mice bearing HCT116 or HT29 human tumour xenografts were randomised into treatment groups to avoid any bias and ensure inter-group consistency, and then treated by oral gavage with either the MCT vehicle (10 ml/kg), 1 mg/kg PD 0325901, 100 mg/kg GDC-0941 or the combination of 1 mg/kg PD 0325901 and 100 mg/kg GDC-0941 once daily for 14 days. Tumour volume was monitored by calliper measurement using the equation *a* ^2^×*b*/2, where *a* is the smallest measurement and *b* the largest. Data are presented as median relative tumour volumes (RTV), where the tumour volume in each mouse on the initial day of treatment (day 0) is assigned an RTV value of 1. The time to RTV3 and RTV4 for each individual tumour was calculated based on a standard point to point curve with 1000 segments using GraphPad Prism software (CA, USA). Mann Whitney U tests were used to compare the different groups, i.e. the control *versus* each treatment group, the single agents *versus* each other, and each agent *versus* their combination. Differences with a p value <0.05 were considered statistically significant.

### [^18^F]-FLT PET Studies

Before treatment and after 2 days of treatment as described above, mice bearing HCT116 human tumour xenografts (7–9 mice/group) were anaesthetised, cannulated *via* their tail vein and placed in the prone position on a custom-made heated bed, which held three mice at once, within a MOSAIC PET scanner (Philips, Eindhoven, NL). [^18^F]-FLT was obtained from PETNET (PETNET, Nottingham, UK), and radioactivity levels were measured using a Capintec well counter (Capintec, NJ, USA). Approximately 10 MBq of [^18^F]-FLT was administered intravenously and a 1 hour dynamic PET scan was performed, consisting of ten 1 minute, six 5 minute and two 10 minute time frames. Mice were recovered after scanning and continued to receive treatment for the remainder of the study. Data were analysed using Imalytics software (Philips, Aachen, Germany); a 3D region of interest was drawn around the tumour and the standardised uptake value (SUV) was calculated by dividing the tissue concentration (MBq/ml) by the injected dose (MBq)/g body weight. Mean SUV values were then plotted against time, and the area under the curve (AUC) and the percentage change in the area under the curve relative to the baseline scan was calculated. Paired t tests were used to compare the day 2 *versus* the baseline SUV AUC values for each individual mouse within each treatment group and differences with a p value <0.05 were considered statistically significant.

## Results

### Pharmacokinetics and pharmacodynamics of PI3K and MEK inhibitors, as single agents and in combination, in HCT116 human tumour xenografts

**Figure 1:**
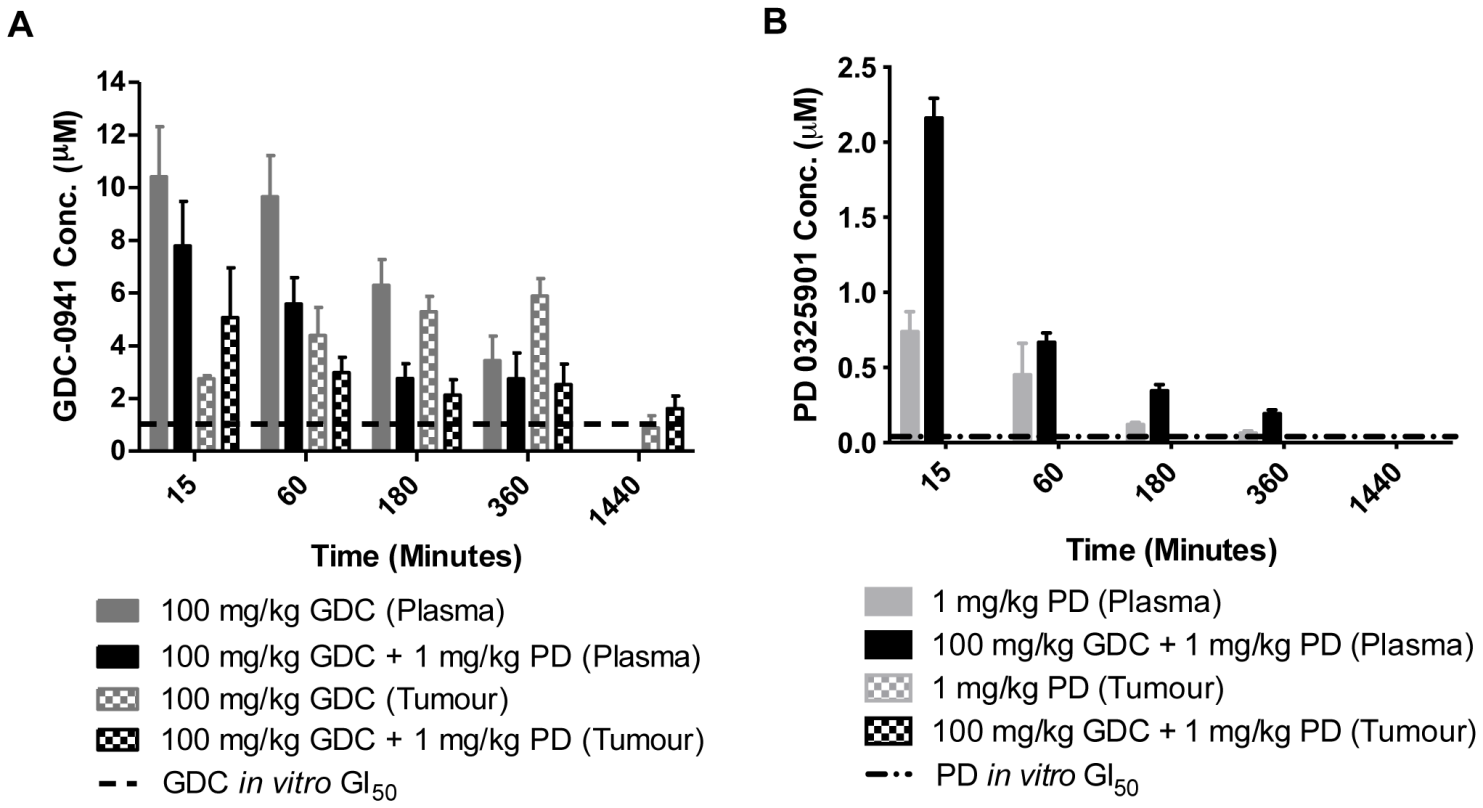
Plasma and tumour concentrations of GDC-0941 and PD 0325901 from mice bearing human tumour xenografts. Plasma and tumour concentrations of the PI3K inhibitor GDC-0941 (GDC) (**A**) and the MEK inhibitor PD 0325901 (PD) (**B**) measured by HPLC in samples from HCT116 tumour xenograft-bearing mice at the indicated time points after a single p.o. dose of either 100 mg/kg GDC-0941 alone, 1 mg/kg PD 0325901 alone or the combination of 1 mg/kg PD 0325901 and 100 mg/kg GDC-0941. Data are presented as the mean concentration from 3 mice in each group ± standard error. The horizontal dashed line indicates the *in vitro* GI_50_ concentration (previously determined in [38]).

A single dose PK/PD study was performed using the PI3K inhibitor GDC-0941 and the MEK inhibitor PD 0325901, as single agents and in combination, in HCT116 human tumour xenograft-bearing mice, over a time course of 0.25–24 hours. The concentrations of the drugs in the plasma and the tumour tissue were measured using HPLC (Figure 1 and Table 1). Concentrations of GDC-0941 and PD 0325901 in the plasma decreased over 24 hours after a single dose of inhibitor (Figure 1). A 10-fold increment in GDC-0941 dose, i.e. 100 *versus* 10 mg/kg, achieved plasma AUC values that were 7-fold higher. In the case of PD 0325901, a comparable dose increase, i.e. 10 *versus* 1 mg/kg, resulted in a 15-fold increase in plasma AUC (Table 1). Concentrations of GDC-0941 and PD 0325901 in the tumour were more variable, and levels of PD 0325901 were undetectable in the plasma at 24 hours (limit of detection: <100 nM) after treatment with 1 mg/kg and in tumour tissue at all time points (limit of detection: <0.4 nmoles/g of tumour material or <100 nM in tumour homogenate diluted four-fold). Tumour GDC-0941 AUC values (Table 1), indicate that there was a 15-fold increase in AUC following a 10-fold increase in dose.

**Table 1:** Plasma and tumour tissue concentration AUC values following GDC-0941 and PD 0325901 administration.

	PD 0325901 AUC (µM.min)		GDC-0941 AUC (µM.min)	
Drug dose	Plasma	Tumour	Plasma	Tumour
1 mg/kg PD 0325901	83±24^a^	-	-	-
10 mg/kg PD 0325901	1296±125^b,c^	1082±232^d,e^	-	-
10 mg/kg GDC-0941	-	-	324±178	364±130^g^
100 mg/kg GDC-0941	-	-	2361±677	5422±962^h,i^
1 mg/kg PD 0325901+10 mg/kg GDC-0941	164±51	-	393±162	396±144^j^
1 mg/kg PD 0325901+100 mg/kg GDC-0941	189±12^a^	-	1356±46	3196±183^h^
10 mg/kg PD 0325901+10 mg/kg GDC-0941	2399±641^b^	399±72^d,f^	380±88	124±51^g,j^
10 mg/kg PD 0325901+100 mg/kg GDC-0941	3248±666^c^	1638±27^e,f^	3529±1929	2649±861^i^

Plasma and tumour tissue concentrations of the PI3K inhibitor GDC-0941 and the MEK inhibitor PD 0325901 were measured by HPLC in samples from HCT116 tumour xenograft-bearing mice over 24 hours after a single p.o. dose of 10 or 100 mg/kg GDC-0941 or 1 or 10 mg/kg PD 0325901, alone and in combination (Figure 1). The area under the curve (AUC) was calculated and data are presented are the mean AUC ± standard deviation. Significant differences between groups are denoted by superscript letters; ^a,c,d,f^ p<0.01, ^b,g,j^ p = 0.04, ^e,h^ p = 0.01, ^i^ p = 0.02.

Interestingly, the plasma AUC data suggests that there can be a modest increase in the concentrations of PD 0325901 in the plasma following concomitant dosing with GDC-0941 (Table 1), and although this difference was limited (2–3 fold), there was a consistent and statistically significant difference between the AUC of PD 0325901 when dosed alone at 1 or 10 mg/kg PD 0325901, or concomitantly with 100 mg/kg GDC-0941 (p <0.01). GDC-0941 administration also appeared to have an effect on the tumour retention of PD 0325901, where it could be measured (i.e. after 10 mg/kg PD 0325901), as there was again a statistically significant difference between the tumour AUC following dosing with 10 mg/kg PD 0325901 alone or concomitantly with 100 mg/kg GDC-0941 (p = 0.01). However, in contrast to the plasma data, following combination treatment tumour concentrations of both PD 0325901 and GDC-0941 were lower than those following single agent treatment, when a significant difference was observed. Thus the enhanced anti-tumour activity observed with the combination of the MEK and PI3K inhibitors (see below) was not due to a pharmacokinetic interaction resulting in increased tumour drug levels. Overall, the PK data demonstrate that there were no marked pharmacokinetic interactions (i.e. >3-fold change in AUC) when GDC-0941 and PD 0325901 were given in combination.

After a single dose of 100 mg/kg GDC-0941, alone or in combination with 1 mg/kg PD 0325901, concentrations of GDC-0941 in the plasma and the tumour tissue consistently exceeded that of the *in vitro* GI_50_ value of 1081 nM (previously determined in [38]) over 6 hours (Figure 1A). Similarly, after a single dose of 1 mg/kg PD 0325901, alone or in combination with 100 mg/kg GDC-0941, concentrations of PD 0325901 in the plasma consistently exceeded that of the *in vitro* GI_50_ value of 21 nM [38] over the first six hours; however, levels were undetectable in the plasma at 24 hours and in the tumour tissue at all time points (Figure 1B). Nevertheless, as the limit of detection for PD 0325901 in both plasma (<100 nM) and tumour tissue (<0.4 nmoles/g) was greater than the *in vitro* GI_50_ value (21 nM), the PK data do not necessarily indicate that pharmacologically active drug concentrations were not achieved.

**Figure 2:**
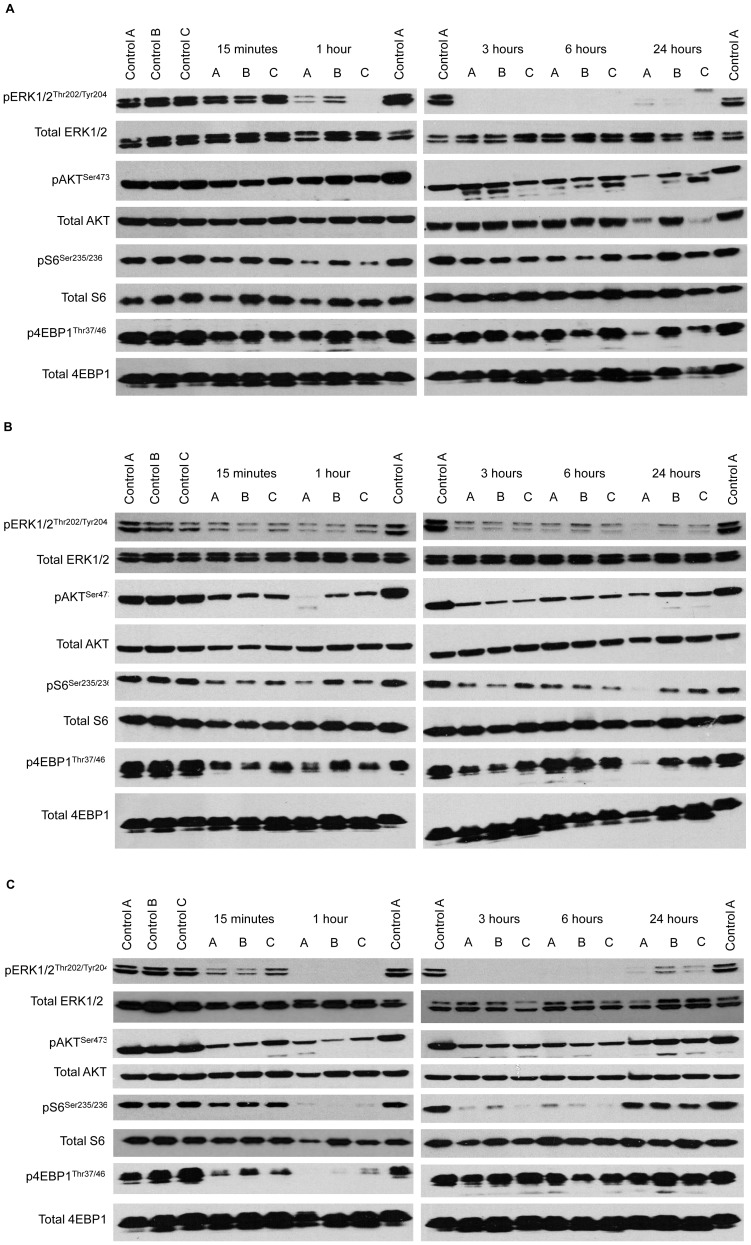
Pharmacodynamic analysis of the *in vivo* effect of PD 0325901 and GDC-0941 on signal transduction. Effects on the MAPK and PI3K/AKT signal transduction pathways after HCT116 tumour xenograft-bearing mice were treated with a single p.o. dose of either 1 mg/kg of the MEK inhibitor PD 0325901 alone (**A**), 100 mg/kg of the PI3K inhibitor GDC-0941 alone (**B**) or the combination of 1 mg/kg of the MEK inhibitor PD 0325901 and 100 mg/kg of the PI3K inhibitor GDC-0941 (**C**). After 0.25–24 hours, tumours were removed and lysates subjected to electrophoresis, followed by Western blotting using the indicated phospho-specific antibody. Blots were then stripped and re-probed with the corresponding total protein antibody to confirm equal protein loading. A–C represents samples from the three mice in each treatment group.

Although, following 1 mg/kg PD 0325901, concentrations were below the limit of detection in the tumour (<0.4 nmoles/g), this dose was able to reduce (1 hour) and completely ablate (3 and 6 hours) the phosphorylation of ERK1/2, with very little recovery by 24 hours. As expected, there was no marked effect of PD 0325901 on AKT, S6 or 4EBP1 phosphorylation (Figure 2A). GDC-0941 at 100 mg/kg was sufficient to cause a reduction in the phosphorylation of AKT, S6 and 4EBP1 over the time course studied, although the reduction was incomplete and the extent of inhibition varied within groups (Figure 2B). Interestingly, there was also a reduction in the phosphorylation of ERK1/2 following a dose of 100 mg/kg GDC-0941. The combination of 1 mg/kg PD 0325901 and 100 mg/kg GDC-0941 caused earlier complete inhibition of ERK1/2 phosphorylation, compared to treatment with the single agent MEK inhibitor, and greater inhibition of S6 and 4EBP1 phosphorylation compared to treatment with the single agent PI3K inhibitor (Figure 2C). However, there was no marked difference between the inhibition of AKT phosphorylation with combination compared to the single agent PI3K inhibitor treatment.

### Efficacy of PI3K and MEK inhibitors, as single agents and in combination, in HCT116 and HT29 human tumour xenografts

**Figure 3:**
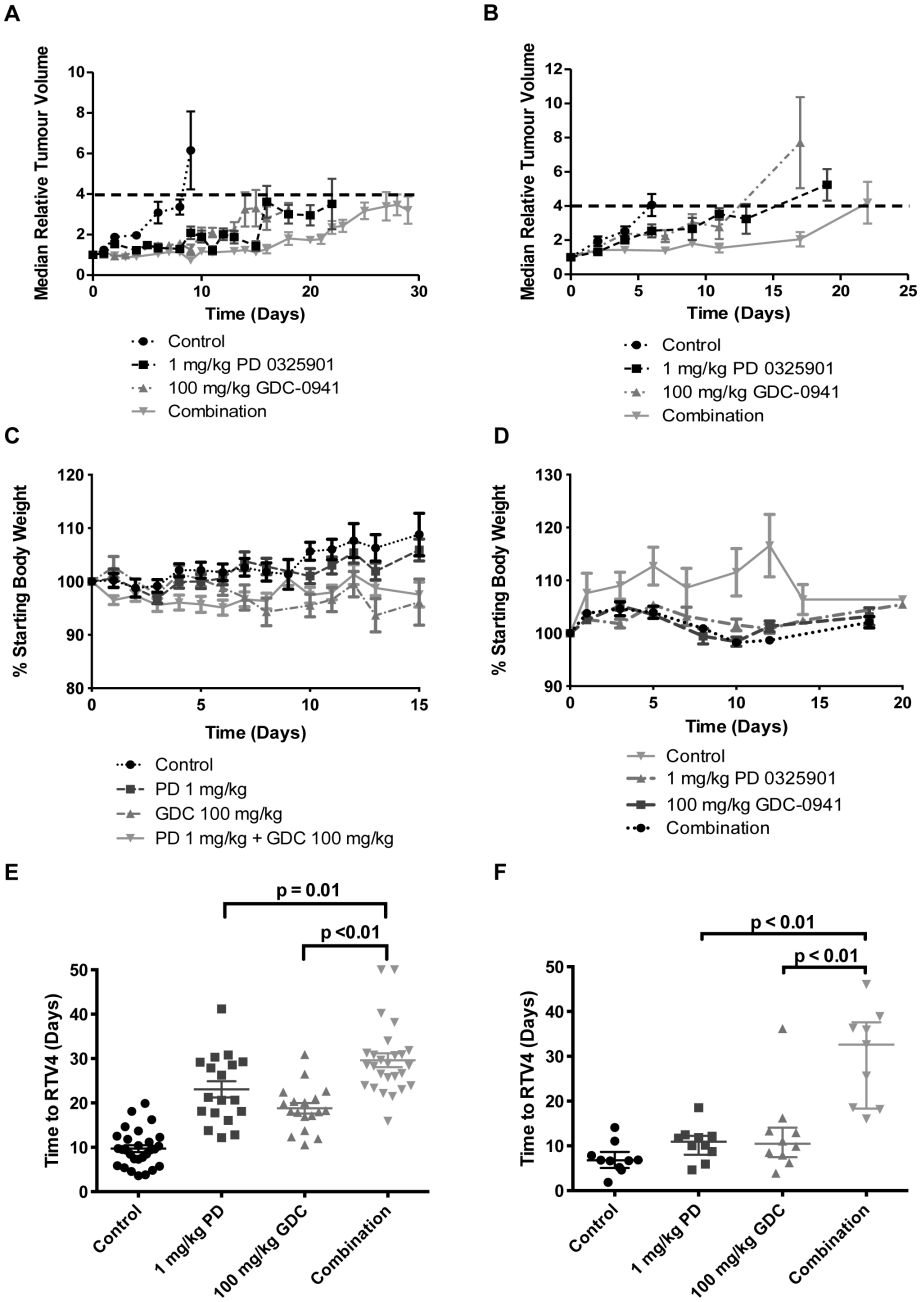
Efficacy and tolerability of GDC-0941 and PD 0325901 in mice bearing human colorectal tumour xenografts. HCT116 (**A, C, E**) and HT29 (**B, D, F**) tumour xenografts were treated with either vehicle control, 1 mg/kg of the MEK inhibitor PD 0325901 and 100 mg/kg of the PI3K inhibitor GDC-0941 alone, or 1 mg/kg of the MEK inhibitor PD 0325901 and 100 mg/kg of the PI3K inhibitor GDC-0941 in combination, p.o. once daily for 14 days. **[A–B]** Tumour growth curves: Data are presented as the median relative tumour volume (RTV), where the growth is calculated for each tumour relative to its size on day 0. Points represent the median of the 10 mice in each group. The dashed line shows the point at which tumours reached four times the initial volume (RTV4). **[C–D]** Effects on body weight: Data are presented as a percentage of starting body weight. Points represent the mean of the 10 mice in each group ± standard error. **[E–F]** Time taken for xenografts to reach four times the initial volume (time to RTV4): Data are presented as the time taken by each individual tumour in each group to quadruple in size, and lines to represent the mean of the mice in each group ± standard error. P values are given where the combination is significantly different from either agent alone (p≤0.05).

Based on the results of the PK/PD study, the efficacy of 100 mg/kg of the PI3K inhibitor GDC-0941 and 1 mg/kg of the MEK inhibitor PD 0325901 given orally, as single agents and in combination, was assessed in HCT116 and HT29 human tumour xenograft-bearing mice (Figure 3). The individual doses of the PI3K and MEK inhibitors were chosen to be equi-active, in order to mirror the *in vitro* conditions under which synergy had been demonstrated previously in these cell lines [38]. In this study, mice were treated daily for 14 days and tumour volumes were measured three times a week. Figures 3A and 3B demonstrate that treatment with 100 mg/kg GDC-0941 and 1 mg/kg PD 0325901, alone and in combination, caused tumour growth delay compared to vehicle-treated control tumours, and that growth delay was greater with the combination. Additionally, body weight was monitored daily to assess the tolerability of the therapy, and both single agent and combination treatments were found to be non-toxic, i.e. body weights did not drop below 90% of the starting weight (Figures 3C and 3D).

The time for the tumours to quadruple in size (time to RTV4) was calculated (Figures 3E and 3F and Table 2), and statistical analyses using a Mann-Whitney test revealed that there was a significant difference between vehicle-treated control tumours and the combination group (p<0.01), and between the single agent inhibitor and the combination groups (p≤0.01), in both HCT116 and HT29 tumour xenograft models. Additionally, there was a significant difference between vehicle-treated control tumours and the single agent inhibitors in the HCT116 tumour xenografts (p<0.01), but not in the HT29 tumour xenografts at the 5% level (p = 0.06).

**Table 2:** Efficacy of GDC-0941 and PD 0325901 in mice bearing human colorectal tumour xenografts.

Tumour xenografts		HCT116		HT29	
Treatment	Calculation	Mean ± SD	Median ± IR	Mean ± SD	Median ± IR
**Control**	**Time to RTV4**	10±4	9±5	7±4	7±2
	**Time to RTV3**	8±4	8±5	6±3	5±2
**1 mg/kg PD 0325901**	**Time to RTV4**	23±8	21±11	11±4	11±3
	**Time to RTV3**	19±7	18±12	10±4	10±4
**100 mg/kg GDC-0941**	**Time to RTV4**	19±5	18±4	13±9	10±5
	**Time to RTV3**	15±4	16±5	11±9	10±6
**1 mg/kg PD 0325901+100 mg/kg GDC-0941**	**Time to RTV4**	30±8	28±7	30±11	33±18
	**Time to RTV3**	27±8	25±6	27±11	30±18

Time taken in days for HCT116 and HT29 tumour xenografts to reach three or four times their initial volume (time to RTV3 or RTV4) when treated with either vehicle control, 1 mg/kg PD 0325901 and 100 mg/kg GDC-0941 alone, or 1 mg/kg PD 0325901 and 100 mg/kg GDC-0941 in combination, p.o. once daily for 14 days. Data are presented as the mean time to RTV3 or RTV4 for the mice in each group ± standard deviation (SD) and the median RTV3 or RTV4 for each group (± interquartile range (IR)).

### [^18^F]-FLT PET scanning on day 2 as a surrogate response biomarker of PI3K and MEK inhibitor efficacy as single agents and in combination in HCT116 human tumour xenografts

**Figure 4:**
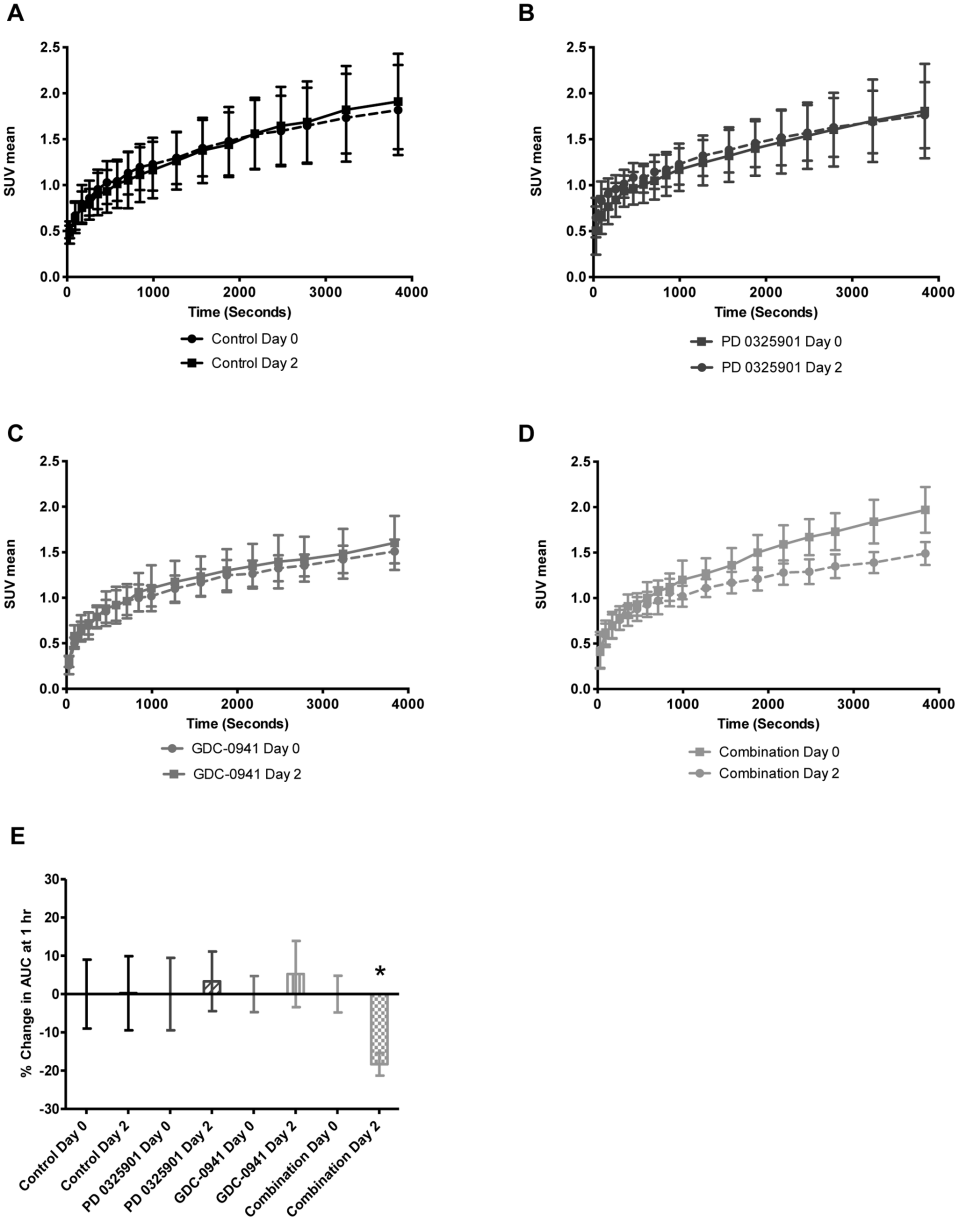
[^18^F]-FLT uptake in human tumour xenografts before and after treatment with GDC-0941 and PD 0325901. [**A–D**] HCT116 tumour xenograft [^18^F]-FLT uptake over the 1 hour dynamic PET scan at baseline and after treatment with either vehicle control (**A**) 100 mg/kg of the PI3K inhibitor GDC-0941 alone (**B**), 1 mg/kg of the MEK inhibitor PD 0325901 alone (**C**) or the combination of 1 mg/kg of the MEK inhibitor PD 0325901 and 100 mg/kg of the PI3K inhibitor GDC-0941 (**D**), p.o. once daily for 2 days. Data are presented as the mean standardised uptake value (SUV), where the tissue concentration is calculated relative to injected dose and body weight. Points represent the mean of the 5–7 mice in each group ± standard deviation. [**E**] Percentage change HCT116 tumour xenograft [^18^F]-FLT uptake at 1 hour before and after treatment with either vehicle control or the combination of 1 mg/kg PD 0325901 and 100 mg/kg GDC-0941, p.o. once daily for 2 days. Data are presented as the percentage change in the area under the curve (AUC) relative to the corresponding baseline AUC, derived from Figures A–D, and bars represent the mean of the 5–7 mice in each group ± standard error. * denotes that the [^18^F]-FLT SUV AUC in the combination treated group were significantly different after 2 days treatment compared to baseline (p<0.01).

It has been proposed that [^18^F]-FLT PET can be used as a surrogate biomarker for tumour response to therapy, and dynamic PET scans were therefore performed after two days of treatment, at which time there were no significant differences in tumour volume between the control or any of the treated groups. Unfortunately, [^18^F]-FLT uptake by HT29 tumours was low and this tumour could not be used for [^18^F]-FLT PET studies. In contrast, HCT116 tumours were [^18^F]-FLT avid and Figures 4A–C show that there were no differences after 2 days in [^18^F]-FLT tumour uptake after treatment with control vehicle, 1 mg/kg PD 0325901 alone or 100 mg/kg GDC-0941 alone, compared to baseline. However, there was a significant decrease in [^18^F]-FLT HCT116 tumour uptake after 2 days of PI3K/MEK inhibitor combination treatment (Figure 4D).

Based on the data in Figures 4A–D, the percentage change in the area under the [^18^F]-FLT SUV *versus* time curve (AUC) in HCT116 tumours was calculated for each individual mouse. Figure 4E shows that there was no significant difference (p = 0.95) between [^18^F]-FLT uptake at baseline and after 2 days of treatment in the control or single agent PD 0325901- or GDC-0941-treated mice. In contrast, there was a statistically significant decrease of 18% in the tumour [^18^F]-FLT uptake after 2 days in the PI3K/MEK inhibitor combination treated mice (p<0.005). These data demonstrate that changes in [^18^F]-FLT uptake precede effects on tumour volume, and that [^18^F]-FLT PET is a valid early surrogate response biomarker for detecting the improved efficacy of combined PI3K and MEK inhibitor treatment.

## Discussion

With some notable exceptions, e.g. imatinib in the treatment of chronic myeloid leukaemia and vemurafenib *BRAF*-mutant melanoma, single agent clinical activity with targeted therapies is modest, presumably due to the presence of multiple driver genetic lesions and the rapid development of resistance mechanisms. Combinations of targeted therapies are therefore being widely investigated. However, in developing optimal combinations, conventional clinical trial methodology has significant limitations as the large number of drugs, patient numbers required and the time taken for response and survival endpoints to be reached precludes the timely evaluation of all potential combinations. Consequently, surrogate response biomarkers, such as [^18^F]-FLT PET, are being investigated in an attempt to generate early proof-of-concept data for the activity of specific combinations. The studies described here have shown that the combination of the PI3K inhibitor GDC-0941 with the MEK inhibitor PD 0325901 is more efficacious *in vivo* than either single agent given at the same doses, and importantly that the increased efficacy of the combination treatment correlates with a subsequent decrease in tumour [^18^F]-FLT uptake measured by PET after just 2 days of treatment.

As reported elsewhere [2], [3], [4], [5], [6] and confirmed here, single agent GDC-0941 was non-toxic and induced tumour growth delay at 100 mg/kg in both the HCT116 and HT29 colorectal tumour xenograft models. However, both HCT116 and HT29 tumours re-grew upon termination of dosing, indicating that GDC-0941 is cytostatic rather than cytotoxic, which is consistent with previous *in vitro* growth inhibition and cytotoxicity studies [38].

The efficacy of GDC-0941 has previously been shown to be associated with decreased AKT and S6 phosphorylation [2], [3], [4], [5], [6], [39], an association that was confirmed here, as there was a decrease in the phosphorylation of AKT and S6 after treatment with 100 mg/kg GDC-0941. Interestingly, in contrast to *in vitro* studies in HCT116 and HT29 cells [38], there was also a decrease in the phosphorylation of 4EBP1 and ERK1/2 phosphorylation *in vivo* after treatment with 100 mg/kg GDC-0941. In particular, the decrease in pERK1/2 levels is in contrast to previous reports that PI3K inhibition may cause the opposite effect as a result of the cross-activation of the MAPK pathway [40]. However, a previous study has demonstrated that dual specificity phosphatase 6 (DUSP6), which dephosphorylates ERK 1/2, can be regulated by the PI3K/mTOR pathway [41], and thus suggests a mechanism for decreased pERK in response to PI3K inhibition. Furthermore, target inhibition does not necessarily correlate directly with efficacy, and O'Brien and colleagues have demonstrated that there was no marked tumour growth inhibition of *KRAS* mutant MDA-MB-231 breast tumour xenografts despite pronounced inhibition of AKT phosphorylation [5].

In the studies reported here, levels of GDC-0941 in the tumour and the plasma over 6 hours were found to greatly exceed the *in vitro* GI_50_ value of GDC-0941 (previously determined in [38]), and drug was still detectable in the tumour 24 hours after a single dose at 100 mg/kg. The pharmacokinetic data presented here also show that the AUCs of GDC-0941 in the plasma and tumour were approximately linearly dose-dependent as levels changed 7-fold and 15-fold, respectively, following a 10-fold increase in dose from 10 to 100 mg/kg.

PD 0325901 was also non-toxic and predominantly cytostatic as a single agent, generating tumour growth delay at 1 mg/kg in both HCT116 and HT29 colorectal tumour xenografts. The data presented here are consistent with previous studies where doses of PD 0325901 ranging from 1.6 mg/kg up to the maximum tolerated dose of 25 mg/kg caused dose-dependent tumour growth delay, stasis and in many cases complete regression in a variety of human and murine tumours [10], [11], [13], [14]. In addition to inhibition of ERK1/2 phosphorylation, one study reported a corresponding decrease in cyclin D1, an upregulation of p27 and decreased phosphorylation of Rb, which resulted in decreased cell proliferation as detected by Ki67 staining [13]. These reported effects of PD 0325901 on ERK1/2 phosphorylation are consistent with the PD data presented in this study, where a marked decrease in the phosphorylation of ERK1/2 was observed after treatment with 1 mg/kg PD 0325901. There was also a small decrease in p4EBP1 and pS6, which may due be to convergence between the MAPK and PI3K/mTOR pathway, as a previous study has demonstrated that MEK inhibition can inhibit S6 and 4EBP1 phosphorylation via the Erk-RSK1-mTOR pathway [42].

Pharmacokinetic analyses revealed that levels of PD 0325901 in the tumour and the plasma greatly exceeded the *in vitro* GI_50_ concentration (previously determined in [38]) for 6 hours following a single dose of 10 mg/kg PD 0325901, and concentrations of PD 0325901 in the plasma were linearly dose-dependent, as levels changed 15-fold following a 10-fold increase in dose from 1 to 10 mg/kg. Furthermore, there were no major pharmacokinetic interactions between GDC-0941 and PD 0325901 (i.e. a>3-fold change in AUC), and the lack of any major interaction is consistent with clinical data for the PI3K inhibitor GDC-0941 in combination with the MEK inhibitor GDC-0973 [43].

In pre-clinical models, combinations of PI3K and MEK inhibitors have consistently shown improved efficacy compared to either single agent alone, causing striking regressions in some cases, in a range of human tumour xenograft and mouse models [6], [12], [17], [18], [19]. For example, the combination of 100 mg/kg GDC-0941 and 6.3 mg/kg PD 0325901 caused regression of AN3CA endometrial and H2122 NSCLC tumour xenografts, compared to a modest tumour growth delay with either single agent alone [6]. This improved activity is consistent with the results presented here as the combination of 100 mg/kg GDC-0941 and 1 mg/kg PD 0325901 was non-toxic, and caused tumour stasis and marked tumour growth delay in the *KRAS* and *PIK3CA* mutant HCT116 and the *BRAF* and *PIK3CA* mutant HT29 colorectal tumour xenografts, respectively, an effect that was significantly greater than either single agent at the same dose (p≤0.01).

In HCT116 tumours, the combination of 100 mg/kg GDC-0941 and 1 mg/kg PD 0325901 was shown to inhibit both the MAPK and PI3K pathways, and enhance the inhibition of ERK1/2, S6 and 4EBP1 phosphorylation, compared to either agent alone. Previous studies have not only reported an inhibition of ERK1/2, AKT and S6 phosphorylation, but have also observed effects on downstream determinants of proliferation and apoptosis, such as a decrease in cyclin D1 and Mcl-1, and an increase in Bim1 accumulation and caspase 3 cleavage [6], [12], [17], [18], [19]. Multiple previous studies have demonstrated that these enhanced effects upon combination treatment are due to convergence between the MAPK and PI3K pathways thus activating common downstream targets such as the transcription factors, FOXO [44], [45], [46] and c-Myc [47], [48], and the pro-apoptotic protein BAD [49], [50], [51], [52], [53]. Furthermore, *in vivo* combination studies have demonstrated that, whereas the phosphorylation of components of the MAPK and PI3K pathways was restored within 72 hours, effects on downstream markers of proliferation and apoptosis were sustained for over 72 hours, suggesting that intermittent dosing of combinations of PI3K and MEK inhibitors may be preferable [12], [19]. Indeed, it has been reported that non-continuous dosing on every 3^rd^ or 4^th^ day with high doses of the PI3K inhibitor GDC-0941, in combination with the MEK inhibitors PD 0325901 or GDC-0973, resulted in marked tumour growth inhibition, and potentially reduced toxicity [6], [19].

To investigate the potential utility of PET scanning as an early surrogate biomarker of tumour response to PI3K and/or MEK inhibitor therapy, PET scanning has been incorporated into efficacy studies in a small number of pre-clinical and clinical studies. For example, [^18^F]-FDG PET has been shown to be a surrogate marker of sensitivity to PI3K inhibition by NVP-BEZ235 and NVP-BKM120 in human HNSCC (FaDu) and mouse mammary (EMT6) 3D tumour spheroids *in vitro* [54], and of response following 100 mg/kg LY294002, 10 mg/kg PF-04691502 or 35 mg/kg NVP-BEZ235 in colorectal, lung and ovarian tumour xenografts and/or mouse models *in vivo* [12], [17], [55]. Similarly, [^18^F]-FDG PET has been shown to be of value in monitoring the activity of the MEK inhibitor GDC-0973 in combination with the BRaf inhibitor vemurafenib in A375 and vemurafenib-resistant A375R1 melanoma xenografts [56].

The PET radiotracer [^18^F]-FLT also represents a promising proof of concept anti-proliferative PD and surrogate response biomarker for PI3K and/or MEK inhibitor therapy. [^18^F]-FLT PET can measure anti-proliferative effects, as it is a thymidine analogue whose accumulation in cells is determined by the expression and activity of thymidine kinase 1 and specific nucleoside transporters, which are under the control of S phase cell cycle regulators [13], [14], [20], [21], [22], [23], and has been shown to correlate with other markers of proliferation [24], [25], [26], [27], [28], [29]. Dynamic [^18^F]-FLT PET scans were therefore incorporated into the HCT116 efficacy studies described in this paper. The tumour uptake of [^18^F]-FLT was monitored over 1 hour at baseline and on day 2 of treatment with the PI3K inhibitor GDC-0941 and the MEK inhibitor PD 0325901, alone and in combination. As has been reported by other studies, [^18^F]-FLT uptake by the HT29 tumours was low [57], [58], [59], and thus tumours derived from this cell line were unsuitable for [^18^F]-FLT PET studies.

The day 2 *versus* pre-treatment dynamic PET scans showed that there was no significant difference in [^18^F]-FLT tumour uptake in HCT116 xenograft-bearing mice treated with drug vehicle or with either single agent, whereas there was a significant decrease in [^18^F]-FLT tumour uptake after PI3K/MEK inhibitor combination treatment, which correlated with the enhanced efficacy observed later in the study. There have been no published pre-clinical or clinical studies measuring [^18^F]-FLT uptake after PI3K and MEK inhibitor combination treatment. There are previous reports that the PI3K inhibitors GDC-0941 or NVP-BEZ235, or the MEK inhibitor PD 0325901, given as single agents caused significant decreases in [^18^F]-FLT uptake as early as 18, 24, 48 or 120 hours, associated with subsequent tumour growth inhibition in a variety of human tumour xenograft models [13], [14], [30], [39]. However, these studies used doses at or close to the single agent MTD which would not be tolerated in combination. In the study presented here, decreased [^18^F]-FLT uptake following combination therapy preceded HCT116 tumour growth inhibition, suggesting that [^18^F]-FLT PET could be used as an early surrogate response biomarker for combined PI3K and MEK inhibitor treatment. Clinical trials involving combinations of PI3K and MEK inhibitors should therefore be extended to include the use of [^18^F]-FLT PET, in parallel to other common proliferation markers, as a biomarker of early response to combination treatment.

Overall, these studies confirm that dual targeting of PI3K and MEK can induce marked tumour growth inhibition *in vivo*, and that this anti-tumour effect can be predicted by measuring [^18^F]-FLT uptake at baseline and after 2 days of treatment. Pharmacodynamic analyses following the combination of the PI3K inhibitor GDC-0941 and the MEK inhibitor PD 0325901 revealed that increased efficacy is associated with an enhanced inhibition of the phosphorylation of ERK1/2, S6 and 4EBP1, compared to that observed with either single agent, and maintained inhibition of AKT phosphorylation. Together these results suggest that in studies of PI3K and MEK inhibitor combinations [^18^F]-FLT PET can be used as an early proof of concept PD and surrogate response biomarker for detecting enhanced anti-proliferative and antitumour effects in a pre-clinical setting, and therefore warrants further testing in clinical trials.

## Acknowledgments

The authors are very grateful to Paul Bevan, Dagmar Ewald, Carola Mala and Wolfgang Schmalix, Wilex AG, Munich, and Colin Stubberfield, UCB Celltech, Slough, for informative and stimulating discussions. Also, the authors are indebted to Celine Cano, Bernard Golding and Roger Griffin for supervising the synthesis of GDC-0941.
